# COVID-19 with Hypoxic Respiratory Failure

**DOI:** 10.5811/cpcem.2020.7.48793

**Published:** 2020-07-16

**Authors:** Miguel A. Martinez-Romo, Shahram Lotfipour, C. Eric McCoy

**Affiliations:** University of California, Irvine Medical Center, Department of Emergency Medicine, Orange, California

**Keywords:** Coronavirus Disease 2019, Coronavirus, COVID-19, ground-glass opacities, chest computed tomography

## Abstract

**Case Presentation:**

We describe an elderly male presenting to the emergency department with shortness of breath that progressed to hypoxic respiratory failure. Radiography and computed tomography findings were suggestive of coronavirus disease 2019 (COVID-19).

**Discussion:**

We review the clinical presentation of COVID-19 and its complications. We also describe the characteristic presentation of COVID-19 on imaging. Our case illustrates the hallmark findings of bilateral and peripheral ground-glass opacities of COVID-19.

## CASE PRESENTATION

**Image 1:**
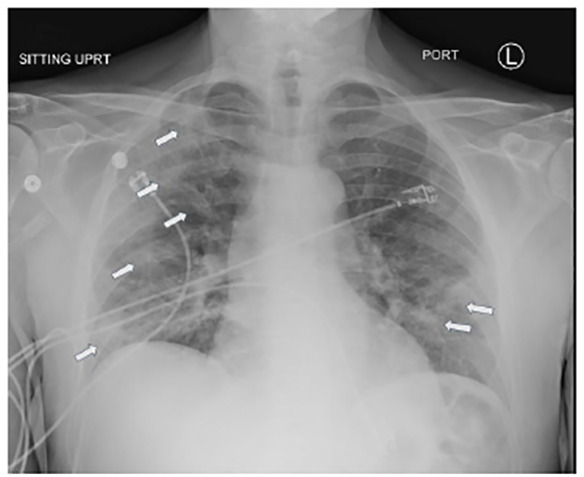
Radiograph demonstrating multifocal patchy ill-defined opacities (arrows) in bilateral lung fields, suggestive of atypical/viral pneumonia.

**Image 2:**
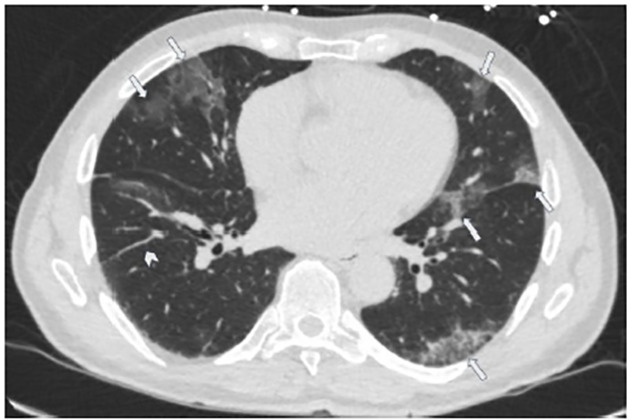
Chest computed tomography (axial image) demonstrating multifocal peripheral bilateral ground glass opacities (arrows) with interlobular septal thickening (arrowhead) and mild peribronchial thickening, suggestive of infectious/inflammatory airway disease which can be seen in the setting of COVID-19.

**Image 3:**
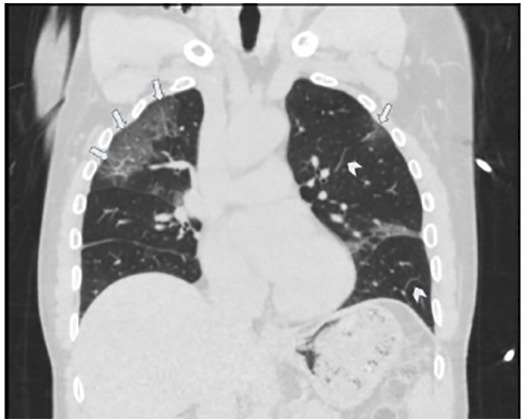
Chest computed tomography (coronal image) demonstrating multifocal peripheral bilateral ground glass opacities (arrows) with interlobular septal thickening (arrowheads) and mild peribronchial thickening, suggestive of infectious/inflammatory airway disease which can be seen in the setting of COVID-19.

A 70-year-old male with a history of hypertension and chronic kidney disease presented to the emergency department (ED) with cough, fevers, and worsening shortness of breath for two weeks. He saw his primary-care physician a week prior and received azithromycin and oseltamivir and was also tested for coronavirus disease 2019 (COVID-19), which was negative. Initial vitals were temperature 38.7°Celsisus, blood pressure 176/87 millimeters of mercury, respirations of 22 breaths per minute, and oxygen saturation of 86% on room air. His exam was significant for tachypnea and diffuse crackles bilaterally. Despite non-invasive oxygenation, he progressed to hypoxic respiratory failure and required intubation. Chest imaging revealed multifocal peripheral bilateral ground-glass opacities suggestive of COVID-19. (Images 1–3) He was admitted to the intensive-care unit and subsequently tested positive for COVID-19.

## DISCUSSION

Severe acute respiratory syndrome coronavirus 2 causes COVID-19.^1^ The virus was first described in China in 2019 as the cause of a cluster of severe cases of viral pneumonia.^2^ The disease spread globally and was declared a pandemic on March 11, 2020.^3^ The clinical presentation of COVID-19 is non-specific and includes fever, cough, fatigue, myalgias, shortness of breath, sore throat, and gastrointestinal symptoms.^4^ Complications include acute respiratory distress syndrome, septic shock, respiratory failure, and death.^4^ In a study from China, computed tomography (CT) was 86.2% sensitive for COVID-19, while radiograph was 59.1% sensitive.^4^ The hallmark findings of COVID-19 on CT are bilateral and peripheral ground-glass and consolidative pulmonary opacities,^5^ which this patient had. Other findings include linear opacities, “crazy-paving” pattern (area of ground-glass opacification with interlobular septal thickening and intralobular lines), the “reverse halo” sign (area of ground-glass opacification with a ring of dense consolidation), local patchy shadowing, bilateral patchy shadowing, and interstitial abnormalities.^4^,^5^

**CPC-EM Capsule**

What do we already know about this clinical entity?

Coronavirus disease 2019 (COVID-19) has a spectrum of clinical presentations, from asymptomatic or mild viral symptoms, to respiratory distress, respiratory failure, severe disease, and death.

What is the major impact of the image(s)?

We present the classic presentation of COVID-19 on chest radiography and computed tomography, which can assist providers in making a diagnosis.

How might this improve emergency medicine practice?

Recognizing COVID-19 on imaging studies can help providers increase their index of suspicion, given the variable speed and availability of confirmatory testing.
